# Steroid-resistant bilateral facial nerve palsies, ophthalmoparesis, and multilevel thoracic radiculopathy as immune effector cell-associated late-onset neurotoxicity after cilta-cel CAR-T therapy: a case report and review of similar cases

**DOI:** 10.3389/fneur.2026.1835346

**Published:** 2026-06-29

**Authors:** James Reid Johnson, Tanya Gupta, Michelle Tavcar, Alex Glynn, Peter Hedera

**Affiliations:** 1 Department of Neurology, School of Medicine, University of Louisville, Louisville, KY, United States; 2 School of Medicine, University of Louisville, Louisville, KY, United States; 3 Division of Hematology Oncology, James Graham Brown Cancer Center, University of Louisville, Louisville, KY, United States; 4 Kornhauser Health Sciences Library, University of Louisville, Louisville, KY, United States

**Keywords:** bell’s palsy, cilta-cel CAR-T, IEC-NP, immune effector-cell late onset neurotoxicities, ophthalmoplegia

## Abstract

The use of chimeric antigen receptor T-cell (CAR-T) therapy has become more widespread in recent years, most commonly for hematologic malignancies. This therapy can be potentially associated with neurotoxic side effects. We present a patient with refractory multiple myeloma who developed diplopia, bilateral upward gaze limitation, abducens palsy, bilateral facial nerve palsy, and thoracic sensory radiculopathy following a course of CAR-T therapy, which were suspected to represent delayed immune effector cell-associated nerve palsies (IEC-NPs). His symptoms progressed despite oral and intravenous steroids. Subsequent intrathecal methotrexate and systemic cyclophosphamide resulted in acute symptomatic improvement and eventual resolution of diplopia and gradual recovery of extraocular movements and facial nerve palsies.

## Introduction

Treatments using chimeric antigen receptor T cells (CAR-T) have transformed the prognosis of hematologic malignancies, especially in relapsed/refractory multiple myeloma (RRMM). Despite their remarkable effectiveness, these therapies can be accompanied by unique toxicities, both acute and delayed after treatment. Acute toxicities most commonly include cytokine release syndrome (CRS) and immune effector cell-associated neurotoxicity syndrome (ICANS). Neurologic symptoms with delayed presentation related to CAR-T treatment have become a more commonly recognized phenomenon and are known as immune effector cell-associated neurotoxicities, including nerve palsies (IEC-NPs), most commonly affecting cranial nerve VII, and also a parkinsonian phenotype (IEC-PKS) with prominent extrapyramidal symptoms (1–4).

Facial palsies tend to be unilateral; however, there have been several individual case reports and series describing IEC-NP as causing bilateral facial palsies (5–8). Some of these patients with facial nerve palsy have had additional neurologic symptoms, including other cranial nerve palsies, phrenic nerve palsies, acute inflammatory demyelinating and axonal polyradiculoneuropathies, axonal sensorimotor neuropathies, and other radiculopathies (9–11). Recently, Lim et al. have published a series of cases of patients with CAR-T-induced immune effector cell-associated late-onset neurotoxicities, including 15 patients with IEC-NPs and 9 patients with IEC-PKS (12). Our case describes a patient with IEC-NP with multiple cranial neuropathies and multilevel thoracic polyradiculopathy who was successfully treated with a unique treatment regimen that contributes to the literature on emerging treatment strategies for IEC-NPs, particularly cases resistant to steroids.

## Case description

### Multiple myeloma and other past medical history

Our patient was initially diagnosed in December 2017, when he presented with back pain, pancytopenia, an M-protein spike of 0.4 mg, lambda light chain elevated to 10,000, and Bence Jones proteinuria. A bone marrow biopsy performed shortly after showed 80–90% plasma cells, and a bone survey showed diffuse marrow changes with CT-confirmed lytic lesions. The patient was started on dexamethasone initially and later transitioned to his first-line treatment of cyclophosphamide, bortezomib, and dexamethasone (CyBorD). He completed eight cycles of treatment without significant events. Evaluation after completing this treatment showed a decrease in lambda light chain to 30, no M spike, and 1% bone marrow plasma cells, achieving a very good partial response (VGPR). Due to this, he was evaluated to proceed with an autologous stem cell transplant. He underwent stem cell mobilization and apheresis with 6.07 × 10 ^6 CD34 + cells collected, and T0 for his transplant took place on 7 September 2018 with a cell dose of 3.07 million. A 90-day bone marrow biopsy revealed no evidence of disease, and the patient continued on maintenance Revlimid. However, he required dose reductions and treatment interruptions due to episodes of pancytopenia and dental implant infections. Due to continued cytopenias, a repeat bone marrow biopsy was performed in August 2022, which showed 27% plasma cells in the marrow. This was performed in conjunction with repeat light chains, serum protein electrophoresis (SPEP), and immunofixation electrophoresis (IFE), which showed lambda light chains of 998.5, kappa light chains of 1.8, and no M-spike. Due to this, he was transitioned to second-line therapy with daratumumab, Pomalyst, Velcade, and dexamethasone. His laboratory work initially showed that he was responding well to treatment, but unfortunately, his lambda light chains increased again along with worsening cytopenias in May 2023. Due to this, he started on third-line therapy with daratumumab, carfilzomib, and dexamethasone. After this regimen change, his light chains downtrended again, and no M-spike was noted. Due to this, he was recommended to undergo a second transplant. He then transferred his care to our academic center and was seen in the clinic for evaluation of a possible second autologous transplant. After discussion, the decision was made for him to proceed with talquetamab therapy instead, with the goal of leaving open the potential for B-cell maturation antigen (BCMA)-directed CAR-T therapy in the future if he responds well, especially since he had not been exposed to BCMA therapy previously. He then started talquetamab on 10 June 2024. However, the patient had multiple adverse effects, including dry mouth, taste changes, and weight gain, and discontinued treatment after two cycles in September 2024. During this time, he had been undergoing workup for Carvykti CAR-T. His marrow on 13 December 2024 was without a significant plasma cell population, and SPEP/IFE showed no monoclonal protein, along with normal free light chain levels. Due to this, he achieved a stringent complete response (sCR), and no further bridging therapy was needed. However, due to his high risk of recurrence, it was recommended that the patient proceed with anti-BCMA CAR-T therapy, Carvykti.

Other known medical conditions prior to CAR-T therapy included hypogammaglobulinemia treated with regular intravenous immunoglobulin (IVIG) infusions, with the last infusion occurring 20 days prior to CAR-T infusion; chronic kidney disease stage 3b secondary to focal segmental glomerulonephritis; and hypertension. He did not have a prior history of tobacco, alcohol, or illicit drug use.

### CAR-T treatment and initial symptoms

He underwent line placement on 4 February 2025 and apheresis of T cells on 6 February 2025 without complication. He underwent conditioning with fludarabine and cyclophosphamide (Flu/Cy) followed by Carvykti on 31 March 2025, defined as day t = 0. On day t + 7, he had a fever of 101 °F. He was admitted to the hospital for workup of neutropenic fever from days t + 7 to t + 10, with a maximum temperature (Tmax) of 103.3 °F on day t + 8, and was treated with empiric antibiotics, although the infectious workup was negative. The fever improved with a single dose of tocilizumab and was believed to be grade 1 cytokine release syndrome. He never experienced symptoms consistent with ICANS. His peak absolute lymphocyte count was 9.7×10^9^/L measured on day t + 11.

On day t + 18, he perceived a new sensation of “heaviness” on the left side of his face without objective weakness and then developed bilateral facial palsy, first on the left side on day t + 20 and then on the right side on day t + 22. He was admitted to the hospital and underwent a traumatic, non-diagnostic lumbar puncture. He was treated with IV dexamethasone 10 mg q8h for 3 days. Furthermore, he was discharged home with oral steroids, specifically prednisone 60 mg with a taper of 10 mg daily, starting on t + 25 and completed on day t + 32; however, he showed minimal improvement in facial weakness. He had significant eye dryness bilaterally, dysarthria, and difficulty eating due to mouth weakness resulting from the facial palsies.

He developed diplopia on day t + 36 and was found to have bilateral vertical gaze palsy and bilateral abducens nerve palsies. On day t + 42, he developed numbness to light touch across his chest and back in a T2–T8 dermatomal pattern. He had been treated with steroids since symptom onset on day t + 18 without improvement in symptoms and was readmitted to the hospital on day t + 44.

### Examination

**Figure 1:**
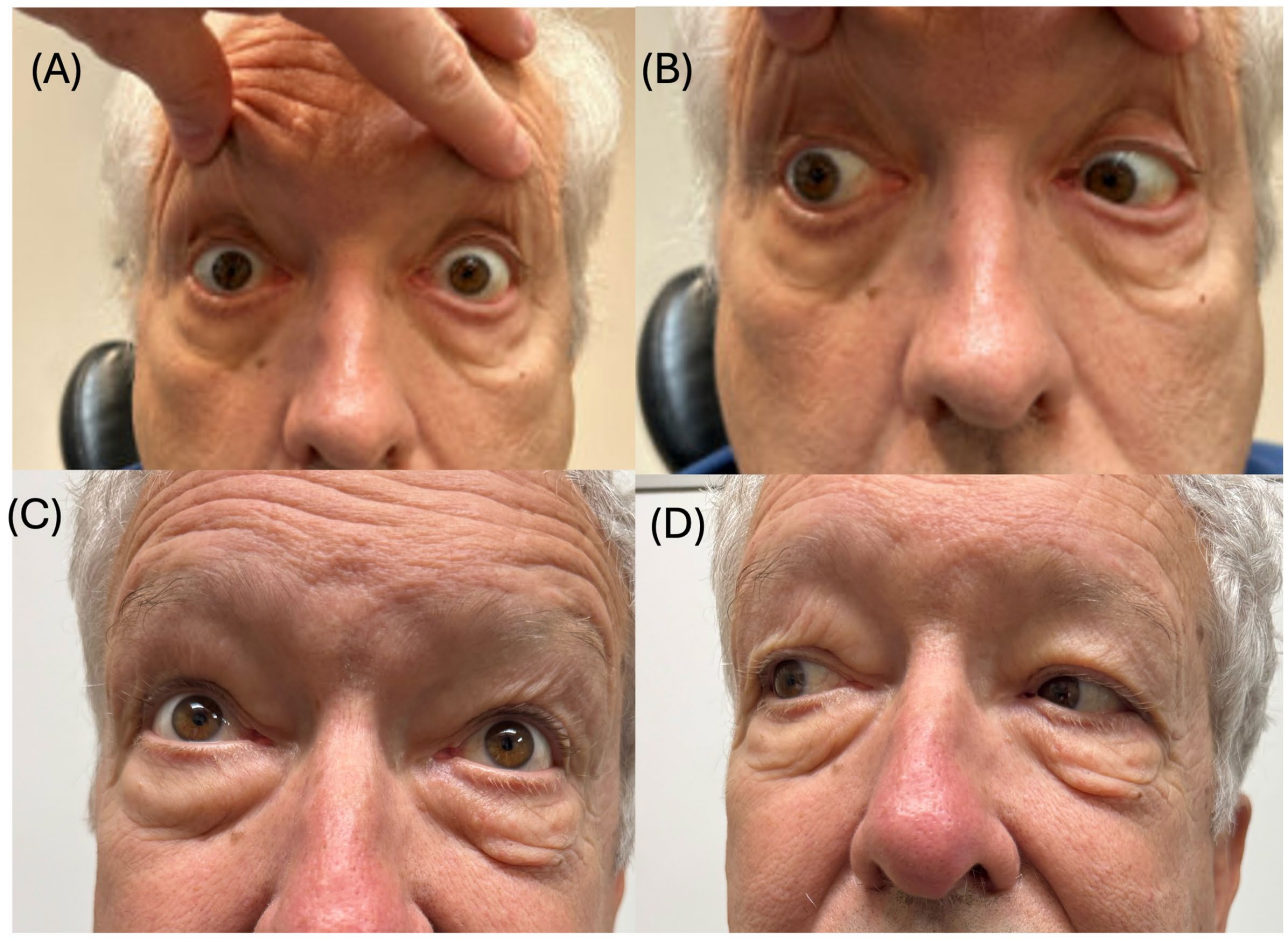
**(A,B)** Pre-treatment gaze palsies; **(A)** attempted supraduction, with inability to look upward; **(B)** attempted rightward lateral gaze, showing grossly limited right abduction, and subtle weakness in right adduction. **(C,D)** Post-treatment improvements in gaze palsies, images taken on t + 318; **(C)** attempted upward gaze, not fully resolved but approximately 50% improvement compared with initial presentation; **(D)** right lateral gaze, with complete resolution of prior palsies, with the patient able to almost fully abduct and adduct the right eye. The patient consented to allowing the above photos to be used for articles. For this figure, boxes are labeled as **A** in the top left box, **B** in the top right box, **C** in the bottom left box, and **D** in the bottom right box.

Examination on day +44 showed dysarthria due to weakness of the orbicularis oris. He had abnormal extraocular movements with an inability to look upward bilaterally beyond the midline position (Figure 1, Box A). Additionally, the left eye had a 25% reduction in abduction, adduction, and downward gaze. The right eye had a 50% reduction in abduction and a 25% reduction in downward gaze (Figure 1, Box B). The pupils were equal, round, and reactive to light; there were no deficits in visual fields or visual acuity, and accommodation was intact.

Facial nerves were affected bilaterally, with grade 5 facial nerve palsy on the right side and grade 6 palsy on the left side. He was only able to close his right eye and raise the right corner of his mouth (to less than 50% of normal) with maximum effort. Facial sensation was intact bilaterally. The rest of the cranial nerves were normal.

The motor examination for all other major muscle groups was normal, with a negative Romberg sign. Reflexes were normal, except for mild hyporeflexia graded 1 + in the left patellar reflex. The sensory examination revealed subjective circumferential absence of sensation to light touch and pain in the T2–T8 dermatomes over his chest and back. Gait and coordination examinations were unremarkable.

### Diagnostic assessment

The differential diagnosis of his new cranial neuropathies included CAR-T-induced neurotoxicities, infectious processes including Lyme disease and other opportunistic bacterial, mycobacterial, fungal, and viral infections, other inflammatory/paraneoplastic processes, leptomeningeal carcinomatosis, and progression of his multiple myeloma.

During his first admission for complaints of bilateral facial weakness, he underwent magnetic resonance imaging (MRI) of the brain without contrast, which did not show any acute abnormality, and a lumbar puncture. Cerebrospinal fluid (CSF) studies showed elevated red blood cells (>20,000), and flow cytometry showed predominant red blood cells, consistent with a traumatic tap. CSF cell count showed elevated total nucleated cells and protein, which were non-diagnostic given the traumatic tap Supplementary Table 1. The total CSF immunoglobulin G (IgG) amount was within normal limits, but the IgG synthesis rate and the IgG index were elevated. Other CSF studies performed to assess known causes of bilateral facial nerve palsies included negative IgM and IgG Lyme antibodies, normal angiotensin converting enzyme, and no oligoclonal bands. Other infectious studies, including a common meningitis PCR panel and cultures for bacteria, acid-fast bacilli, and fungi, showed no growth at 5 days, 6 weeks, and 4 weeks, respectively. Serum paraneoplastic/autoimmune encephalitis paneling from the Mayo Clinic revealed a very mildly elevated anti-GAD IgG level of 0.05; however, the detected level was still low, and he had no clinical symptoms to support autoimmune encephalitis.

On the second admission for new-onset of diplopia and paresthesia, he underwent MRI of the thoracic spine without contrast, which showed a bulging disk of T8–T9 causing 25% stenosis of the thecal sac. MRI of the cervical spine without contrast did not show significant compression or signal change. A second lumbar puncture was performed, and flow cytometry showed no populations of plasma cells but showed 78.43% lymphocytes; 75.47% of the total population consisted of T cells (CD5+). Other studies of the CSF, including cell count, were negative for an infectious process.

The bilateral cranial nerve palsies and paresthesia were attributed to suspected neurotoxicity caused by CAR-T treatment due to the temporal relationship of his symptoms, CSF evidence of predominant T-cell involvement, diagnostic workup ruling out other infectious or malignant causes, and literature reports of similar presentations with timelines similar to that of our patient. The diagnostic certainty was somewhat limited by the lack of contrast-MRI studies, which were not performed due to the patient’s reduced renal function, and by the patient’s decision not to undergo nerve conduction studies to assess his thoracic radiculopathy.

### Treatment

His treatment initially consisted of both oral and short-course, high-dose IV corticosteroids: IV dexamethasone 10 mg q8h for 3 days on initial presentation of facial nerve palsies, which did not improve symptoms, followed by an oral prednisone taper starting at 60 mg over 8 days. Despite receiving high-dose steroids for 13 days, he developed more troubling and disabling neuropathies. During the second hospital admission, he was placed on IV dexamethasone 10 mg q8h for 5 days. He also received one-time doses of intrathecal methotrexate 12 mg, cytarabine 50 mg, and hydrocortisone 25 mg on day t + 46, and one dose of IV cyclophosphamide 40 mg/kg with IV MESNA 4.5 g on day t + 49. He was discharged from the hospital on day t + 50. At discharge, intravenous steroids were transitioned to oral dexamethasone 20 mg daily, which the patient self-discontinued after a gradual taper, completing cessation on day t + 101.

### Outcome and follow-up

He showed an increase in right lateral gaze and a subjective increase in right facial movements, eye movements, and diplopia on day t + 47 following the intrathecal methotrexate and hydrocortisone dose. His examination on day t + 106 at a follow-up neurology appointment showed almost complete resolution of diplopia and significant improvement in extraocular movements, with near resolution of CN III and CN VI palsies and significant improvement in right CN VII palsy. Left-sided facial paralysis had not improved until 6 months after onset, and on day t + 338, both facial palsies had improved to only grade 2. He had no reported infections or other major side effects attributed to either intrathecal therapy or cyclophosphamide. At his most recent oncology follow-up on day t + 379, he showed no evidence of active myeloma on PET-CT and no evidence of plasma cells in the bone marrow. He has yet to show any signs or symptoms of parkinsonism more than 1 year after treatment.

## Discussion

**Figure 2:**
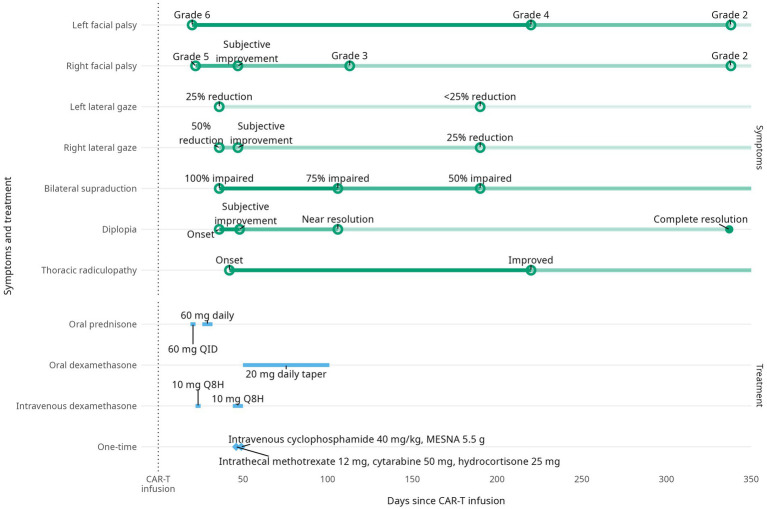
Swimmer plot showing symptom onset and treatments administered.

Our case describes multiple, bilateral cranial neuropathies coexisting with thoracic polyradiculopathies that began approximately 3 weeks after receiving CAR-T treatment, and were suspected to represent delayed immune effector cell-associated nerve palsies (IEC-NPs). Our case is similar to other cases, particularly in that he had multiple cranial neuropathies along with other neurologic symptoms described in the recent review published by Lim et al. The strengths of our case report include a well-documented timeline and examination of progressive cranial neuropathies (Figure 2), along with a unique treatment approach that we believe relieved symptoms and also possibly helped prevent progression of more serious neurologic toxicities.

The exact mechanism of delayed-onset immune-cell effector-mediated neurotoxicities following cilta-cel CAR-T is not well known. Some mechanistic insights were suggested in a case of bilateral facial diplegia following cilta-cel infusion: chemokine-driven trafficking (IP-10/CXCR3, CCR6/CCL20) and direct CAR-T infiltration into the central nervous system, which were likely mediators of neuropathy, as demonstrated through CSF immunophenotyping (7). The direct cytotoxic effects are believed to be due to the “on-target, off-tumor” phenomenon of CAR-T. This is supported by autopsy reports of high-affinity CAR-T cells binding to BCMA-expressing neurons and astrocytes, with evidence of focal gliosis and T-cell infiltration (as shown on immunohistochemistry) of the caudate nucleus in a patient with IEC-PKS (13). Patients in the CARTITUDE-1, −2, and −4 trials with facial nerve palsy were found to have elevated serum IL-10 and increased exposure to IL-10 and IL-2Ra (4).

Lim et al. have expanded our knowledge of reported symptoms associated with late-onset neurotoxicities. They reported 15 patients with IEC-NPs, 11 with isolated facial nerve palsies, 8 with unilateral involvement, and 3 with bilateral involvement. There were four other patients with IEC-NPs and additional neurologic symptoms. One patient developed simultaneous IEC-NP and IEC-PKS. Another patient had orthopnea related to phrenic nerve palsies, pupil-sparing oculomotor nerve palsy, and facial nerve palsy. One patient had multiple cranial neuropathies, including unilateral oculomotor, trigeminal, abducens, and bilateral facial nerve neuropathies, along with severe, diffuse axonal polyradiculoneuropathy. The fourth patient had unilateral facial nerve palsy along with chronic sensorimotor polyneuropathy, radiculopathy, and possible post-synaptic neuromuscular junction disorder that the patient would eventually succumb to. They have reported that the median time to onset of IEC-NP was 21 days. Sidana et al. have published a review of patients with cranial nerve impairment who were enrolled in the CARTITUDE-1, −2, and −4 trials and have found that 21 of the total 332 patients developed cranial neuropathies, with a median time to symptom onset of 22 days, and 12 of 21 had concurrent additional neurologic symptoms/toxicities (4).

The reported treatment responses to delayed neurotoxicities vary based on the phenotype. Lim et al. have reported that 14 of 15 patients with IEC-NPs had complete resolution of symptoms, with a median time to resolution of 57 days. Of the patients who received systemic corticosteroids, 13 of 14 had complete resolution. They reported using additional immunomodulatory agents, including IVIG, for patients with IEC-NPs who had additional multifocal neurologic complaints. Nine patients with IEC-PKS had more variable responses to treatment. Two patients received no treatment; one succumbed to IEC-PKS-related complications, and the other had resolution of symptoms. Three patients were treated with steroids; two had incomplete symptom resolution, and the third succumbed to IEC-PKS-related complications. There were an additional four patients treated with cyclophosphamide, and they all had significant improvements in symptoms. None had died by the time of article publishing.

Our patient’s case is consistent with previously described cases of IEC-NPs with regard to timing and risk factors. He displayed symptoms on day t + 20, which is similar to previously reported cases. His peak absolute lymphocyte count (ALC) after CAR-T was elevated, 9.7 ×10^9^, and this is consistent with Lim et al.’s finding that having a peak ALC of > 3× 10^9^ /L was associated with a significantly higher risk of developing delayed neurotoxicities. Our patient continued to develop cranial neuropathies despite receiving both oral and brief high-dose IV corticosteroids. He received intrathecal methotrexate, hydrocortisone, cytarabine, and systemic cyclophosphamide and had acute improvement in gaze palsy, facial palsy, and diplopia while not developing any other new neurologic symptoms.

There are limitations to our case related to diagnostic uncertainty, as the first lumbar puncture performed was traumatic, gadolinium contrast-enhanced MRIs were unable to be obtained, and no thoracic or limb EMG was performed. However, there was a close association between the onset of new symptoms and the start of CAR-T, a negative workup of other causes of cranial polyneuropathies, and CSF flow cytometry showing predominant T-cell populations. These findings were fully consistent with CAR-T neurotoxicity. Additionally, after the patient was discharged from the hospital on day t + 50, his symptom improvement is documented using outpatient encounters, so it is possible that symptoms may have improved in the time between appointments, which may make our record of symptoms somewhat unreliable.

We believe that our case contributes to the existing literature and supports the consideration of additional immunomodulatory agents in the treatment of IEC-NPs, particularly in cases with additional neurologic symptoms and/or progression despite systemic steroids. The Lim et al. review and expert opinions advocate for the use of steroids for cranial nerve palsies and the addition of IVIG for refractory and/or co-occurring multifocal symptoms (10). The Mayo Clinic also recommended, in IEC-PKS, the use of IVIG and Solu-Medrol as initial treatment and consideration of treatment escalation if there is no response after 3–5 days, to include either intrathecal hydrocortisone and chemotherapy or systemic cyclophosphamide. The use of cytotoxic chemotherapeutic agents has also been used in the treatment of refractory ICANS (14, 15). There have been no known reports of primary IEC-NPs treated with systemic cyclophosphamide and intrathecal hydrocortisone and methotrexate. Our case suggests that the use of additional agents such as cyclophosphamide and/or intrathecal chemotherapies and hydrocortisone may be helpful in rapidly progressive, late-onset neurotoxicity secondary to CAR-T.

In conclusion, we report a unique presentation of IEC-NP with bilateral oculomotor, abducens, and facial nerve palsies along with multilevel thoracic polyradiculopathies, further expanding known peripheral nervous system complications after CAR-T therapy. The response to steroids was minimal, but symptoms improved, and no other symptoms occurred after receiving intrathecal chemotherapy and systemic cyclophosphamide, providing further anecdotal evidence supporting treatment escalation in steroid-resistant immune effector cell nerve palsies.

## Data Availability

The datasets presented in this article are not readily available because of ethical and privacy restrictions. Requests to access the datasets should be directed to the corresponding author.

## Author’s note

The patient consented to the use of his clinical records and photographs of his face for this case report.
