# Supplementary material for: Steroid-resistant bilateral facial nerve palsies, ophthalmoparesis, and multilevel thoracic radiculopathy as immune effector cell-associated late-onset neurotoxicity after cilta-cel CAR-T therapy: a case report and review of similar cases
Source: Front Neurol. 2026 Jun 29;17:1835346. doi: 10.3389/fneur.2026.1835346 (PMC13360426; doi:10.3389/fneur.2026.1835346)
Supplement: Supplementary file 1 [file Table_1.docx]

| Work Up from 1^st^ Admission | | |
| --- | --- | --- |
| Test Name | Result | |
| MRI Brain without Contrast | No evidence of metastasis or abnormal signal in brainstem | |
| CSF Studies | | |
| Test Name | Result | Ref Range |
| CSF Total Nucleated Cells | 44 | 0-5 mm3 |
| CSF Red Blood Cells | 20,592 | 0-5 mm3 |
| CSF Protein | 64 | 10-45 mg/dL |
| CSF Glucose | 89 | 40-70 mg/DL |
| Common Meningitis PCR Panel | neg | neg |
| Bacterial CSF Culture | No growth at 5 days |  |
| Mycobacterial CSF Culture | No growth at 6 weeks |  |
| Fungal CSF Culture | No growth at 4 weeks |  |
| CSF ACE Level | 7 | < 15 U/L |
| Lyme Disease IgM and IgG Immunoblot | Negative | Negative |
| CSF Flow Cytology:  “Predominantly blood. Traumatic Tap” | | |

| Work Up from 2^nd^ Admission | |
| --- | --- |
| Test Name | Result |
| MRI Cervical Spine without contrast | Multilevel degenerative changes, no significant cord impingement |
| MRI Thoracic Spine without contrast | Right paracentral disc protrusion causes 25% stenosis, stable compared to prior PET (2 months prior to CAR-T). No evidence of abnormal cord signal |
| CSF Flow Cytometry | Predominantly T Cells (75.7% CD 5+), no evidence of abnormal Plasma cell populations |
